# The impact of momentary stress on autobiographical memory recall in a self-efficacy intervention

**DOI:** 10.1038/s41598-024-80896-z

**Published:** 2024-12-02

**Authors:** Judith Rohde, Laura E. Meine, Adam D. Brown, Birgit Kleim

**Affiliations:** 1 Department of Adult Psychiatry and Psychotherapy, Psychiatric University Hospital Zurich, Lenggstrasse 31, Zurich, CH-8032 Switzerland; 2 Department of Psychology, University of Zurich, Binzmuehlestrasse 14, Box 1, Zurich, CH-8050 Switzerland; 3 Department of Psychology, New School for Social Research, New York, USA; 4 Department of Psychiatry, New York University School of Medicine, New York, USA

**Keywords:** Autobiographical memories, Momentary stress, Memory recall, Self-efficacy intervention, Ecological momentary assessment, Psychology, Human behaviour, Quality of life

## Abstract

Recalling positive autobiographical memories has been associated with various positive psychological outcomes, including enhanced mental well-being and self-efficacy. Given the known impact of stress on cognitive functioning, we investigated how momentary stress affects the repeated recall of selected autobiographical mastery memories (e.g., memories of overcoming challenges) in a training to enhance self-efficacy. During this one-week digital training, participants (*N* = 54) were asked to recall mastery memories, and we assessed their momentary stress levels, memory vividness, and recall feasibility using Ecological Momentary Assessment. Analyses using linear mixed-effects models showed that participants reported greater difficulty and less vividness in recalling self-efficacy memories during moments of increased stress, whereas feeling relaxed facilitated recall feasibility and vividness. Though participants who experienced less recall difficulty appeared to benefit more, recall difficulty and vividness did not significantly moderate improvements in self-efficacy. While replication in a larger, more diverse sample is indicated, our findings underscore the importance of considering momentary affect in memory-based mental health interventions. Effects may be particularly pronounced when interventions are applied during the early stages of stress, when stress levels are still relatively low, aligning with the strategy of Just-in-Time Adaptive Interventions. Our study also highlights the potential benefits of combining memory recall practices with relaxation-promoting interventions to enhance mental health outcomes.

**Supplementary Information:**

The online version contains supplementary material available at 10.1038/s41598-024-80896-z.

## Introduction

The recollection of autobiographical memories represents events relevant to one’s life and even the individual’s self-identity. Additionally, considerable work indicates that various aspects of the content and characteristics of autobiographical memories also contribute to behaviors and affective processing. For instance, autobiographical memories play a crucial role in decision-making^1–3^ and influence the reinforcing value of physical activity^4^. Apart from this directive role, two other functions appear relevant, which are social, in terms of social bonding, and self, encompassing self-expression and self-continuity^5–7^. There is also an effect of age, in that younger individuals more frequently utilize autobiographical memories for directing future behavior and social bonding. Additionally, females tend to use autobiographical memories more for directing their behavior, while age and gender show no significant differences in the other respective functions^8^.

Furthermore, studies show that selectively recalling positive autobiographical memories may be associated with positive mental health. Recalling positive autobiographical memories appears to consistently activate multiple brain regions^9^, correlating with various positive psychological outcomes. For instance, recalling positive autobiographical memories through reminiscence-based interventions is associated with reduced risk aversion^2^ as well as enhanced mental well-being, positive psychological states, and anticipated pleasure^10,11^. Relatedly, recalling autobiographical self-efficacy memories (e.g., memories of success, overcoming adversity, and challenges) is associated with increased self-efficacy, better social problem-solving, and goal-oriented future thinking^12^. Central to this is the concept of self-efficacy, which refers to individuals’ belief in their ability to overcome challenges and succeed in tasks^13,14^. Self-efficacy may serve as a buffer against stress^15,16^, positively influences psychological resilience^17^, and acts protectively in the context of mental health^18–20^. These benefits underscore the potential of self-efficacy memories for training purposes. For instance, experimentally increasing self-efficacy has been found to enhance the reappraisal of negative memories^21^, improve emotional flexibility^22^, and reduce hopelessness and anxiety, along with enhancing self-efficacy when controlling for baseline self-efficacy^23^.

Building upon these findings, we developed an Ecological Momentary Intervention (EMI) delivering a one-week digital self-efficacy training based on recalling autobiographical self-efficacy memories aiming to enhance self-efficacy and other psychological parameters^22,23^. The idea for using EMI was built on the premise that EMI seamlessly integrates scalable psychological interventions into individuals’ daily lives^24,25^. We combined the digital self-efficacy training with an Ecological Momentary Assessment (EMA), capturing transient variables such as mood and stress^26^. The outcomes of the self-efficacy training and the analyses of EMA parameters have been reported elsewhere^22,23^.

Taking into consideration that acute stress impairs cognitive functions including memory retrieval^27–29^, we were particularly interested in how momentary stress affects repeated recall of a previously retrieved and defined autobiographical self-efficacy memory in a memory-based psychological intervention. Specifically, we examined memory recall feasibility and memory vividness. Additionally, we wanted to gain knowledge if memory recall feasibility and vividness influence the effects of the self-efficacy memory recall intervention. We hypothesized that elevated stress levels render it more difficult to effectively recall self-efficacy memories and that these memories would thus be perceived as less vivid. In contrast, we expected reports of greater feasibility and vividness in moments when participants feel more relaxed. In addition, we probed the underlying mechanism of the intervention by testing whether recall feasibility and memory vividness moderate the observed increases in self-efficacy.

## Results

Participants showed good compliance with, on average, 20 completed self-efficacy trainings including associated EMA (*M* = 19.39, *SD* = 7.04, range = 9–46), resulting in 1047 total data points for the entire sample. No cases had to be excluded.

ICC values reflected a substantial amount of variance due to between-person differences in ratings of feeling stressed (42.62%) and relaxed (29.54%). We therefore set up LMEMs which are more appropriate in this case as they account for the hierarchical structure of the data (data points nested within individuals). Results revealed a significant positive effect of feeling stressed on recall difficulty (*β* = 0.11, *t* = 2.73, *p* = 0.006) and a significant negative effect on memory vividness (*β* = -0.09, *t* = -2.61, *p* = 0.009). When participants felt more stressed than usual, they found it more difficult to recall autobiographical self-efficacy memories, and these memories were perceived as less vivid. Conversely, feeling relaxed showed a significant negative effect on recall difficulty (*β* = -0.18, *t* = -4.76, *p* < 0.001) and a significant positive effect on vividness (*β* = 0.15, *t* = 4.45, *p* < 0.001) with participants reporting less difficulty and greater memory vividness if they felt more relaxed. There was no effect of prompt type in any of the models, indicating no difference of scheduled versus self-triggered prompts on recall difficulty or memory vividness. Detailed model results are shown in supplementary Tables 1–4.

We also tested whether less recall difficulty and greater memory vividness were associated with intervention success, i.e. a greater increase in self-efficacy from pre- to post-intervention.

Although visualization of the data suggested that participants who reported the greatest recall difficulty did not appear to benefit from the intervention (see supplementary Figs. 1 and 2), we did not observe significant interaction effects. Neither average recall difficulty (*β* = -0.47, *t* = -0.96, *p* = 0.341), nor average memory vividness (*β* = 0.15, *t* = 0.31, *p* = 0.760) significantly moderated changes in self-efficacy. See supplementary Tables 5 and 6 for model details.

**Fig. 1:**
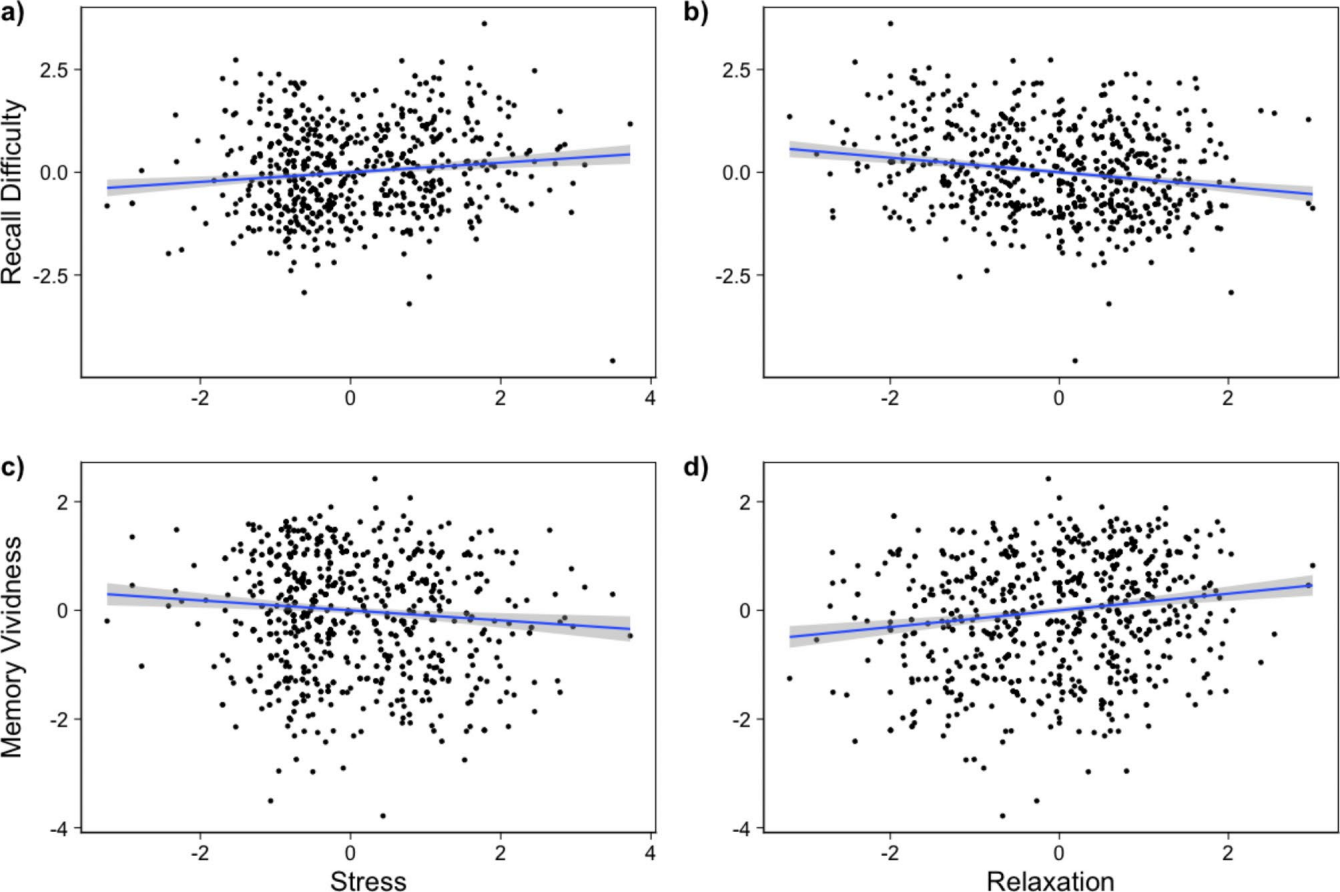
Associations of feeling stressed with memory recall difficulty (**a**) and vividness (**b**) and links between feeling relaxed and recall difficulty (**c**) and memory vividness (**d**). Plots depict within-person standardized data, i.e., the blue line shows the number of within-person SDs the average person’s recall difficulty or vividness change for a one within-person SD increase in stress or relaxation, respectively. The shaded area represents the standard error.

## Discussion

Research indicates that recalling memories may contribute to positive health and well-being outcomes. In particular, a growing body of studies shows that retrieving autobiographical memories of success and achievement (self-efficacy memories) may support several clinically relevant processes^17,20,30–32^. This study aimed to build on existing work related to self-efficacy autobiographical memories by examining states of stress on self-efficacy memory recall. Specifically, we investigated state effects on the feasibility of recalling autobiographical self-efficacy memories and their vividness within a one-week intervention to boost general self-efficacy.

In line with our hypotheses, participants had greater difficulty recalling the memories and perceived them as less vivid in moments when they were more stressed. Feeling more relaxed, on the other hand, showed beneficial effects on recall feasibility and memory vividness. In contrast to our hypotheses, recall feasibility and memory vividness did not significantly moderate improvements in self-efficacy.

This finding does not, however, negate the effectiveness of the intervention itself as participants in the intervention group overall showed significantly greater increases in self-efficacy and related constructs compared to controls (see Rohde et al., 2022, 2023). However, individual and momentary effects of the intervention may nevertheless be context-dependent, and here we investigated the context of stress and found significant effects of moderation, i.e., effects on specific intervention processes and outcomes depending on momentary stress levels. Additionally, other factors such as the content of the memory or the expectations preceding the recall, may also influence the success of the intervention. Since data visualization suggested that participants who experienced more difficulty in recalling self-efficacy memories did not benefit, it may also be that our sample was too small to detect an effect.

The literature discusses challenges in the retrieval of autobiographical memory, including issues such as false memories and age-related memory decline^33–36^. Foundational works on how emotional states influence memory recall have shown that people are more likely to recall information congruent with their current mood. For example, in a positive mood, it is easier to recall positive memories^37–39^. Recent work has demonstrated that mood-congruent memory effects not only enhance the recall of mood-congruent content but may also lead to the formation of false memories, particularly in negative mood states. Additionally, there is growing interest in the neurobiological mechanisms that support these effects, although further research is needed in this area^40^. Given that mastery memories are often positive and related to self-efficacy, momentary stress and the resulting negative or neutral mood^41–43^ may disrupt their recall due to mood-congruent effects, which favor the retrieval of mood-aligned, often negative content. Studies have also highlighted how the emotional content of memories influences their specificity, type, and detail. Retrieval, however, can be hindered by negative or depressed mood^39,44^. Furthermore, acute stress itself is well known to negatively impact cognitive functioning^27^ and specifically hinders episodic memory recall^28,29^. For instance, it has been broadly investigated how stress before or during exams influences performance and how altered levels of glucocorticoids affecting hippocampal function impair memory retrieval. While stress has been shown to enhance encoding and consolidation, especially for emotional experiences, it affects episodic memory retrieval, particularly the controlled retrieval involving prefrontal cognitive control and attention mechanisms. Regarding the effect of stress on cued recall, findings have been mixed, with some studies reporting no significant effect and others indicating impairment. However, overall evidence suggests that stress may impair free recall to a greater extent than cued recall^45^. The influence of stress on repeated and cued recall of autobiographical memories, a common component of psychological interventions, remains relatively unexplored. Our study aimed to address this gap by investigating the impact of stress on the recall of previously retrieved and recorded autobiographical memories. Our findings demonstrate that even when memories are cued and recall is practiced an average of 20 times per week, stress significantly hinders retrieval, thereby impacting study outcomes.

The current study has some limitations. Firstly, our analyses were exploratory, potentially biasing the results. Secondly, our sample was rather small and relatively homogenous in terms of age, origin, and education, limiting the generalizability of our findings. While we took steps to avoid potential bias and ensure robust results (including exclusion of participants with low compliance, tests of model assumptions, and transparent reporting and provision of data and code), and observed consistent effects across models, replication in a larger, more diverse sample would further strengthen confidence in the generalizability and robustness of our findings. Thirdly, we did not assess participants’ initial difficulty in finding and recording autobiographical self-efficacy memories at the outset of the study. Fourth, while we aimed to isolate recall difficulty in our measure of task performance, it is possible that the breathing instructions and the imagination process also contributed to participants’ evaluations. This should be considered when interpreting the results. Future studies may benefit from more targeted questions explicitly distinguishing between recall difficulty and other elements of the task. Moreover, the stress level was measured with limited items, which restricts the depth of our understanding. It would be interesting to investigate more comprehensively whether and at what level stress may be beneficial (e.g., low levels) and when it becomes detrimental, or if this can be applied to memory recall. Finally, we relied on self-assessment and did not involve physiological measures to assess stress states such as cortisol levels or heart rate variability.

Despite these limitations, our study also has several strengths and clinical implications. Using an EMI allowed participants to recall autobiographical self-efficacy memories over one week, integrated into their daily lives. Although the effects were rather small, they point to the importance of momentary affect, in this case stress levels, in memory-based interventions, even if repeated memory recall is involved. This aligns with the principles of Just-in-Time Adaptive Interventions (JITAIs), which emphasize the strategic timing of interventions to maximize their effectiveness^46,47^. Future research could encourage participants to practice memory recall, preferably when they feel relaxed. Combining memory recall practices with mindfulness-based or other relaxation-promoting interventions, especially during the early stages of stress, may result in increased efficacy. This approach aligns with the current trends in precision prevention research, which increasingly recognize the value of timely and context-sensitive interventions, although such designs are still underutilized in many areas^48^.

## Methods

### Participants

From a randomized controlled trial evaluating a self-efficacy training in 93 participants (ClinicalTrials.gov Identifier: NCT05617248), we included the intervention group in this study (*n* = 54, 78% female, 23.72 years (*SD* = 3.16)). Inclusion criteria were a moderate stress level indicated by a score of ≥ 13 on the Perceived Stress Scale^49,50^, current enrollment as a student at a Swiss university, aged between 18 and 29 years, owning a smartphone, and proficiency in the German language. Individuals with a current psychiatric disorder were excluded. The baseline characteristics of the participants in the intervention group showed a mean Beck Depression Inventory-II (Beck, 1996) sum score of 13.96 (*SD* = 8.96) and a mean Perceived Stress Scale sum score of 22.48 (*SD* = 5.88).

The study was approved by the ethics committee of the Faculty of Arts and Social Sciences of the University of Zurich (ethics approval No. 20.4.24) and conducted in accordance with the Declaration of Helsinki. All participants provided electronic informed consent.

## Procedure

Participants received video-based psychoeducation on self-efficacy and instructions to define two autobiographical self-efficacy memories. They were asked to record these memories before starting the intervention. The most frequent memory theme reported was academic success (e.g. “Despite experiencing a prior panic attack and migraine, and thus being inadequately prepared, I successfully navigated the bachelor thesis presentation.”), followed by accomplishments in social and personal domains (e.g. “Despite feeling nervous, I performed my first concert in front of an audience.”, “I had previously sewn women’s clothing. Pakistani men’s Salwar Kameez, however, presented a somewhat greater challenge. Nonetheless, I watched YouTube videos and eventually taught myself. Before long, my first men’s outfit was complete.”) and excelling in sporting events (e.g. “Through courage, determination, and presence, I successfully completed the Engadine Ski Marathon”). Following this, participants engaged in the digital self-efficacy training for one week. The training (EMI) and the momentary state questions (EMA) were administered through the SEMA3 platform, an open-source software application for advanced smartphone surveys^51^. The self-efficacy training sessions were scheduled three times per day during fixed time windows (11:30 a.m.-12:30 p.m., 3:00–4:00 p.m., and 8:30 − 9:30 p.m.) and were no longer accessible if not reacted to within 50 min. After being encouraged to breathe slowly and regularly to enhance focus and facilitate the self-efficacy training, participants engaged in an imagination task designed to gradually lead them to recall one of their two self-efficacy memories. Additionally, participants could self-trigger the self-efficacy training at any time.

Before each training session, EMA assessed current mood states including stress and relaxation levels, introduced with the instruction “Please answer the following questions about how you feel at the moment.”, followed by the questions, e.g., “I feel relaxed.” or “I feel stressed”. The answers were rated on a 7-point Likert scale (1 = not at all to 7 = very much). After each training session, participants were asked two questions: (1) “How did this exercise go in general?” rated on a 7-point Likert scale (1 = easy to 7 = difficult), and (2) “How vivid was the imagination of your memory?” rated on a 7-point Likert scale (1 = not vivid at all to 7 = very vivid).

At pre- and post-intervention, different variables including general self-efficacy using the General Self-Efficacy Scale (GSE; Schwarzer^52^; Tipton & Worthington^53^) were collected. Please see our prior publications for a detailed description of the methods including all other measures, how we determined our sample size, data exclusions, and manipulations^22,23^.

## Data analysis approach

We included both scheduled and self-triggered training sessions in our analysis to make use of all available data. Participants were excluded if they completed less than 25% of scheduled prompts (i.e., less than five) and we excluded surveys completed outside the data collection period (i.e., before day 1 or after day 7). We calculated intra-class correlation coefficients (ICCs) for our variables of interest to gauge the amount of inter-individual versus intra-individual variability. A high ICC indicates considerable differences between participants, suggesting a multilevel model to account for nesting of data points within individuals. We subsequently set up linear mixed effects models (LMEMs) to investigate whether feeling stressed or relaxed immediately prior to memory recall hampers or facilitates it, respectively. Specifically, we tested effects on recall difficulty and memory vividness. The predictor (stressed or relaxed) and outcome (recall difficulty, memory vividness) were within-person standardized so that we could analyze how much the average person’s recall difficulty or vividness changed when their stress or relaxation level increased (see also Wang et al.^54^). We included type of prompt (scheduled/self-triggered) as a covariate because participants may have triggered surveys more often when stressed. Random intercepts were not added as these are zero due to within-person standardization. However, we included random slopes to allow for inter-individual differences in the association between predictor and outcome and added autocorrelations because self-triggered prompts could be closer together in time. Finally, we tested whether recall difficulty and memory vividness moderated change in self-efficacy from pre- to post-intervention. This was investigated with two LMEMs with self-efficacy as the outcome predicted by time (pre/post) and an interaction term of either time x average recall difficulty or time x average memory vividness, including by-participant random intercepts. Here, predictors were z-standardized before inclusion in the model. Analyses were conducted in R, version 4.3.2^55^, using the ‘nlme’ package for LMEMs^56^. Data and code will be made available upon publication on the Open Science Framework: https://osf.io/crx54/.

## Electronic supplementary material

Below is the link to the electronic supplementary material.

Supplementary Material 1

## Data Availability

Data and code have been made publicly available via OSF and can be accessed at: https://osf.io/crx54/?view_only=25ea66227cd943278062b4a7d6ffcf71.
