# Supplementary material for: The impact of momentary stress on autobiographical memory recall in a self-efficacy intervention
Source: Sci Rep. 2024 Dec 2;14:29864. doi: 10.1038/s41598-024-80896-z (PMC11612412; doi:10.1038/s41598-024-80896-z)
Supplement: Supplementary file 1 — Supplementary Material 1 [file 41598_2024_80896_MOESM1_ESM.pdf]

Supplement

The impact of momentary stress on autobiographical memory recall in a self-efficacy intervention

Judith Rohde[1,2 †] *, Laura E. Meine[1,2 †], Adam D. Brown[3,4], Birgit Kleim[1,2]

[1] Department of Adult Psychiatry and Psychotherapy, Psychiatric University Hospital Zurich, Lenggstrasse 31, CH-8032 Zurich, Switzerland

[2] Department of Psychology, University of Zurich, Binzmuehlestrasse 14, Box 1, CH-8050 Zurich, Switzerland

[3] Department of Psychology, New School for Social Research, New York, United States of America

[4] Department of Psychiatry, New York University School of Medicine, New York, United States of America

[†] These two authors contributed equally to this work.

* Corresponding author (e-mail address: judith.rohde@bli.uzh.ch)

Table of Contents

1. *Supplementary Tables* *2*

Supplementary Table 1. Stress and recall difficulty – model details 2

Supplementary Table 2. Stress and memory vividness – model details 2

Supplementary Table 3. Feeling relaxed and recall difficulty – model details 3

Supplementary Table 4. Feeling relaxed and memory vividness – model details 3

Supplementary Table 5. GSE scores by time x recall difficulty – model details 4

Supplementary Table 6. GSE scores by time x memory vividness – model details 4

2. *Supplementary Figures* *5*

Supplementary Figure 1. Moderation effect of average recall difficulty on pre-post change in self-efficacy 5

Supplementary Figure 2. Moderation effect of average vividness on pre-post change in self-efficacy 5

Supplementary Tables

Supplementary Table 1. Stress and recall difficulty – model details

	Fixed Effects				
Predictors	*Estimates*	*SE*	*95% CI*	*T*	*p*
Intercept	0.01	0.04	-0.06 – 0.08	0.24	0.808
Stress level (within-person)	0.11	0.04	0.03 – 0.20	2.73	**0.006**
Prompt type	-0.04	0.07	-0.16 – 0.09	-0.55	0.585
**Random Effects**					
σ^2	0.90				
τ_{00}					
τ_{00}					
ICC	0.04				
N_{id}	54				
Observations	1046				
Marginal R^2 / Conditional R^2	0.013 / 0.056				

Supplementary Table 2. Stress and memory vividness – model details

	Fixed Effects				
Predictors	*Estimates*	*SE*	*95% CI*	*T*	*p*
Intercept	0.02	0.04	-0.05 – 0.10	0.60	0.550
Stress level (within-person)	-0.09	0.03	-0.16 – -0.02	-2.61	**0.009**
Prompt type	-0.07	0.07	-0.20 – 0.06	-1.10	0.273
**Random Effects**					
σ^2	0.93				
τ_{00}					
τ_{00}					
ICC	0.01				
N_{id}	54				
Observations	1046				
Marginal R^2 / Conditional R^2	0.010 / 0.023				

Supplementary Table 3. Feeling relaxed and recall difficulty – model details

	Fixed Effects				
Predictors	*Estimates*	*SE*	*95% CI*	*T*	*p*
Intercept	0.01	0.04	-0.06 – 0.09	0.41	0.684
Feeling relaxed (within-person)	-0.18	0.04	-0.25 – -0.10	-4.76	**<0.001**
Prompt type	-0.05	0.07	-0.18 – 0.08	-0.80	0.426
**Random Effects**					
σ^2	0.90				
τ_{00}					
τ_{00}					
ICC	0.02				
N_{id}	54				
Observations	1046				
Marginal R^2 / Conditional R^2	0.031 / 0.053				

Supplementary Table 4. Feeling relaxed and memory vividness – model details

	Fixed Effects				
Predictors	*Estimates*	*SE*	*95% CI*	*T*	*p*
Intercept	0.02	0.04	-0.06 – 0.09	0.48	0.635
Feeling relaxed (within-person)	0.15	0.03	0.09 – 0.22	4.45	**<0.001**
Prompt type	-0.06	0.07	-0.19 – 0.07	-0.84	0.401
**Random Effects**					
σ^2	0.92				
τ_{00}					
τ_{00}					
ICC	0.01				
N_{id}	54				
Observations	1046				
Marginal R^2 / Conditional R^2	0.024 / 0.036				

Supplementary Table 5. GSE scores by time x recall difficulty – model details

	Fixed Effects				
Predictors	*Estimates*	*SE*	*95% CI*	*T*	*p*
Intercept	24.74	0.90	22.93 – 26.55	27.39	**<0.001**
Time	1.98	0.49	1.00 – 2.96	4.06	**<0.001**
Recall Difficulty	-0.45	0.91	-2.28 – 1.37	-0.50	0.618
Time x Recall Difficulty	-0.47	0.49	-1.46 – 0.51	-0.96	0.341
**Random Effects**					
σ^2	6.43				
$\tau_{00\ id}$	11.89				
N_{id}	54				
Observations	108				
Marginal R^2 / Conditional R^2	0.272 / NA				

Supplementary Table 6. GSE scores by time x memory vividness – model details

	Fixed Effects				
Predictors	*Estimates*	*SE*	*95% CI*	*T*	*p*
Intercept	24.74	0.91	22.91 – 26.57	27.16	**<0.001**
Time	1.98	0.49	0.99 – 2.97	4.03	**<0.001**
Vividness	0.81	0.92	-1.03 – 2.65	0.89	0.380
Time x Vividness	0.15	0.49	-0.84 – 1.14	0.31	0.760
**Random Effects**					
σ^2	6.54				
$\tau_{00\ id}$	12.12				
N_{id}	54				
Observations	108				
Marginal R^2 / Conditional R^2	0.241 / NA				

Supplementary Figures

Supplementary Figure 1. Moderation effect of average recall difficulty on pre-post change in self-efficacy

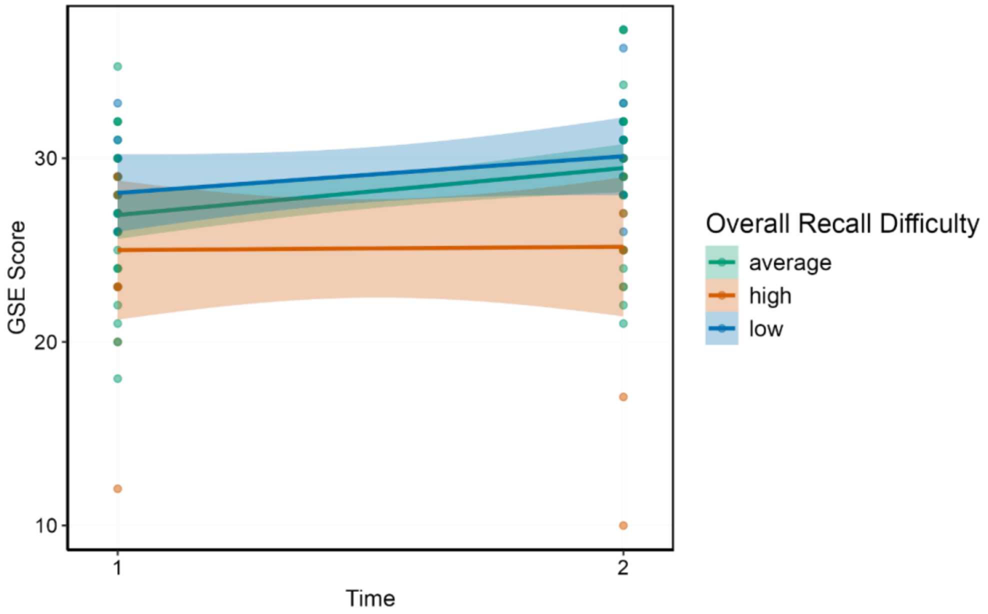

Note. For visualization purposes, we grouped individual data points for recall difficulty into high (values more than one standard deviation above the mean; red), average (values within one standard deviation around the mean; green), and low (values less than one standard deviation below the mean; blue). GSE = general self-efficacy.

Supplementary Figure 2. Moderation effect of average vividness on pre-post change in self-efficacy

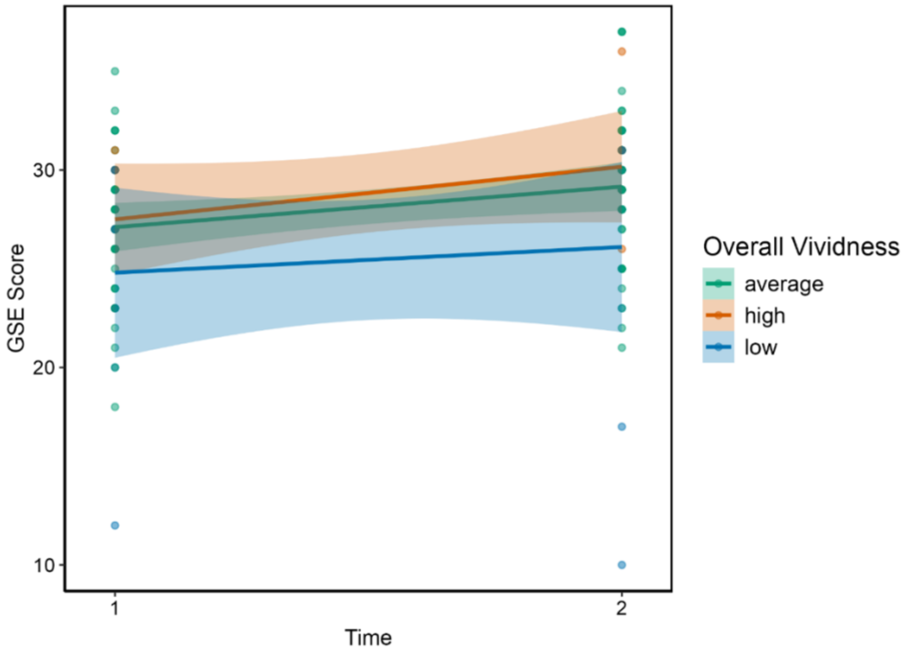

Note. For visualization purposes, we grouped individual data points for vividness into high (values more than one standard deviation above the mean; red), average (values within one standard deviation around the mean; green), and low (values less than one standard deviation below the mean; blue). GSE = general self-efficacy.
